# Automatic Segmentation of Colon in 3D CT Images and Removal of Opacified Fluid Using Cascade Feed Forward Neural Network

**DOI:** 10.1155/2015/670739

**Published:** 2015-03-09

**Authors:** K. Gayathri Devi, R. Radhakrishnan

**Affiliations:** ^1^Dr. N. G. P. Institute of Technology, Coimbatore 641048, India; ^2^Vidhya Mandhir Institute of Technology, Tamilnadu, India

## Abstract

*Purpose*. Colon segmentation is an essential step in the development of computer-aided diagnosis systems based on computed tomography (CT) images. The requirement for the detection of the polyps which lie on the walls of the colon is much needed in the field of medical imaging for diagnosis of colorectal cancer. *Methods*. The proposed work is focused on designing an efficient automatic colon segmentation algorithm from abdominal slices consisting of colons, partial volume effect, bowels, and lungs. The challenge lies in determining the exact colon enhanced with partial volume effect of the slice. In this work, adaptive thresholding technique is proposed for the segmentation of air packets, machine learning based cascade feed forward neural network enhanced with boundary detection algorithms are used which differentiate the segments of the lung and the fluids which are sediment at the side wall of colon and by rejecting bowels based on the slice difference removal method. The proposed neural network method is trained with Bayesian regulation algorithm to determine the partial volume effect. *Results*. Experiment was conducted on CT database images which results in 98% accuracy and minimal error rate. *Conclusions*. The main contribution of this work is the exploitation of neural network algorithm for removal of opacified fluid to attain desired colon segmentation result.

## 1. Introduction

Colorectal cancer occurs either in the colon or in the rectum. Most colorectal cancers initially developed a colorectal polyp, which is growths inside the colon or rectum that may later become cancerous. It affects both men and women mostly above the age of 50. It is the third most common cancer among men after prostate and lung cancer [1]. For women, colorectal cancer is the third most common cancer after breast and lung cancers [2]. Because of this dreadful disease, research in colon segmentation has accelerated only in the last few years. A common approach involved in the segmentation of colon includes the following three steps.

- Air surrounding the body should be removed.
- Air contained in the lungs is masked.
- Segment the colon on the various slices.

But, the above mentioned steps are fundamental and do not provide the desired results in all the scenarios. The process of colon segmentation is a challenging task. Some of the points related to the difficulty in colon segmentation are listed below.

1. The colon is not the only gas filled structure in the abdomen but there are other organs which are having the same intensity values as the colon. Slices adjacent to the colon contain portion of lung which should be removed.
2. The existence of different areas that have the same high CT number (intensity) such as bones, air, and contrast enhancement fluid (CEF) should be removed for proper segmentation of colon.
3. One finds obstructions such as stools, small bowel, polyps, and residual feces in the colon.
4. One finds folding and unfolding of the colon structures such as haustral folds on the borders of colon.
5. Length of the colon and the location of the colon vary for different slices.

These difficulties make the segmentation of colon more complicated specially in situation where an automated algorithm should be implemented.

But, in recent years, certain advanced approaches have been developed to deal with colon segmentation. Most of the segmentation approaches are exploited only on conventional colonoscopy, capsule endoscopy, and barium enema X-ray examination methods. Recently, it is observed that CT colonography uses low dose radiation CT scanning to obtain an interior view of the colon (the large intestine) [3, 4] whereas in endoscopy, insertion of tube in the rectum will affect the privacy of the patient. There is a chance of abdominal pain and to lessen that a muscle-relaxing drug may be injected intravenously to relax the bowel and to reduce the discomfort. This is not the case with CT colonography [5]. The difference between a polyp and fecal material can be revealed easily by the radiologist.

The following points clearly describe the advantages of CT colonography compared to conventional colonoscopy, capsule endoscopy, and barium enema X-ray examination.

- It enables the radiologist to see inside the colon without having to insert a viewing instrument, the colonoscope into the bowel.
- This new minimally invasive test provides both 2D and 3D images that can depict many polyps and other lesions as clearly as when they are directly seen by conventional colonoscopy.
- Elderly patients, especially those who are ill, will tolerate CT colonography better than conventional colonoscopy.
- CT colonography can be helpful when colonoscopy cannot be completed because the bowel is narrowed or obstructed for any reason, such as by a large tumor.
- CT colonography is less costly than colonoscopy.

Thus, in this work, an automatic segmentation method is proposed for colon segmentation from abdominal computed tomography (CT) images. Since not many works are carried out for the segmentation of colon with automated process, this work focuses on developing an efficient automation segmentation approach. The proposed multistage detection method is employed to extract colon based on the colon anatomy information. This may be a rapid and easy diagnosis method of colon at an earlier stage and help doctors for analysis of correct position of each part and detect the colon rectal cancer much easier. Virtual colonoscopy combines axial spiral CT data acquisition of the air-filled and cleansed colon with 3-dimensional imaging software to create endoscopic images of the colonic surface [6]. This paper focuses on the advantages of computer-aided detection (CAD) techniques for the segmentation of colon which will aid the identification of polyps for the detection of colorectal cancer.

In this proposed methodology, the air packets are extracted from the input image by Otsu thresholding method and then the lung slices are extracted from the input image. The extracted lung image was fed as input to the cascaded feed forward neural network for prior training of the partial volume effect region. The extraction of feature such as the intensity and the location were processed by correlating the input slice with its corresponding ground. Finally, the tiny holes and the bowels of the image are removed by slice difference removal method (SLDR). Thus, the main contribution of the work is to improve the results of colon segmentation through cascaded neural network.

*Related Works*. A number of semiautomated and automated methods for the segmentation of colon have been proposed. Bert et al. proposed the automatic segmentation of colon using 3D seeded region growing algorithm [5]. Losnegård et al. [7] proposed a semiautomated method for the segmentation of sigmoid and descending colon and the removal of bowels was performed using fast marching method. The segmentation results were evaluated using the parameter dice coefficient (DC).

Lu and Zhao [8] proposed a method that can remove noncolonic attachments and connect collapsed colonic segments together for the VC examination. The proposed method is mainly composed by a noncolonic attachment classification algorithm and a heuristic connection algorithm. Computer-generated colons are compared with human-generated colons which are manually extracted by two radiologists.

Chowdhury and Whelan [9] propose a fast and accurate method for automatic colon segmentation from CT data using colon geometrical features. After removal of the lung and surrounding air voxels from CT data, labeling is performed to generate candidate regions for colon segmentation. The centroid of the data, derived from the labeled objects, is used to analyze the colon geometry. Other notable features that are used for colon segmentation are volume/length measure and end points.

Taimouri et al. [10] use constrained least-squares filtering (CLSF) technique for preprocessing and both cleansing and stool detection in the colon are done prior to segmentation. Lu and Zhao [11] proposed an improved method for colon segmentation using two widely virtual colon unfolding (VU) techniques, the ray-casting technique and the conformal-mapping technique.

Kilic et al. [12] proposed an automatic three-dimensional computer-aided detection system for colonic polyps. Computer-aided detection for computed tomography colonography aims at facilitating the detection of colonic polyps. First, the colon regions of whole computed tomography images were carefully segmented to reduce computational burden and prevent false positive detection. In this process, the colon regions were extracted by using a cellular neural network and then the regions of interest were determined. In order to improve the segmentation performance of the study, weights in the cellular neural network were calculated by three heuristic optimization techniques, namely, genetic algorithm, differential evaluation, and artificial immune system. Afterwards, a three-dimensional polyp template model was constructed to detect polyps on the segmented regions of interest. At the end of the template matching process, the volumes geometrically similar to the template were enhanced.  Table 1 lists the various methods proposed and the limitations of that method for the segmentation of colon.

The abovementioned section clearly discusses the existing colon segmentation approaches and its limitations which affect the overall performance of the system.

## 2. The Proposed Segmentation Scheme

**Figure 1:**
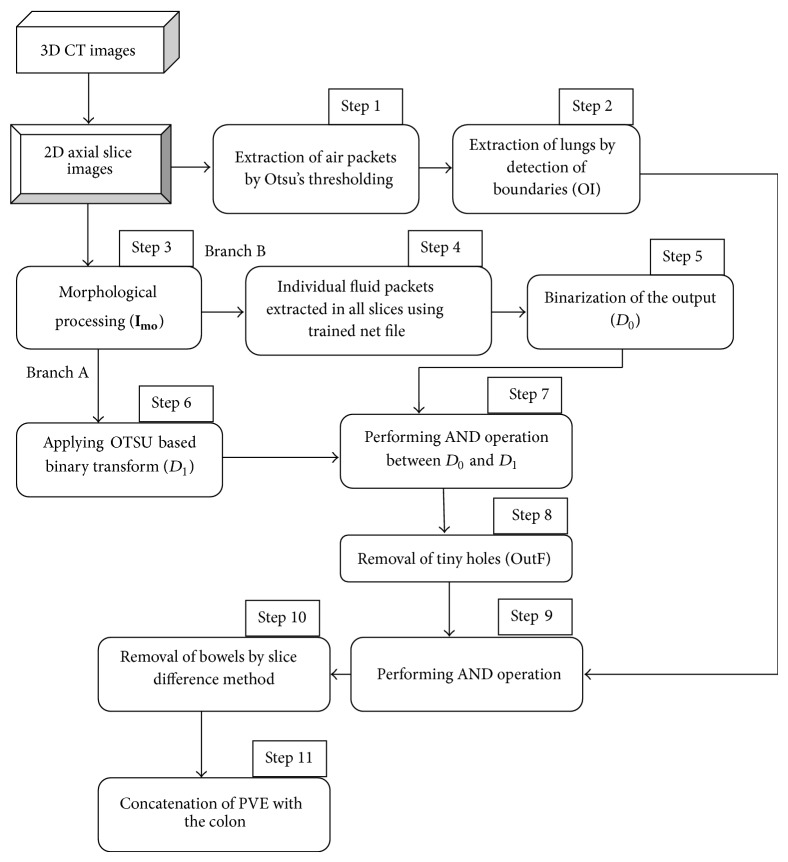
The overview of the proposed method.

The overall block diagram of the proposed method is shown in Figure 1. Abdominal CT image in DICOM format of size 512 × 512 obtained from TCIA imaging Archive link [13] and real time dataset obtained from imaging centre are given as the input to the proposed method. Each dataset consists of millions of voxels with slices ranging from approximately 225 to 650 which makes the processing of million voxels difficult. So, in this approach, seven 2D slices out of 625 torso CT scan were considered as training samples for the automatic segmentation of colon. The proposed approach starts with identification of all the air filled regions in 2D axial slices. In this region, colon and noncolon portion should be classified. These regions contain bowels, small intestine, lungs, and other random noises. Foreground should be separated from the background by removing the air portions that are outside the body of the subject. After the presegmentation process, each segmented component is labeled as one of the five anatomical categories such as colon, small intestine, mixed (colon and small intestine), stomach or lung, and noise. Mixed class and colon categories are considered as positive class. All the others are negative class. The removal of the negative class, which is masking of the lungs, is performed based on the calculation of the area occupied by different boundaries and the removal of the bowels is carried out based on slice difference method.

The procedure adopted for the extraction of fluid filled parts inside the colon was implemented using cascade feed forward neural network. The networks were already trained to handle the partial volume effect which consist of air filled region, fluid filled region, and boundaries (AFB) which interconnect the air-fluid as shown in Figure 4(a). The network will handle the partial volume effect, extract the fluid region, and fill the holes using morphological processing approach. Thus, after the removal of bowels and the random noises, concatenation of the extracted partial volume effect region with the above segmented colon segments provides the final output.

The portions of colon segmented by the proposed algorithm are compared with the manually segmented colon structures segmented using ITK snap software [14]. The performance of the proposed method is evaluated with parameters, namely, accuracy, dice coefficient, sensitivity, and specificity and is compared with adaptive level set and graph cut methods.

**Figure 2:**
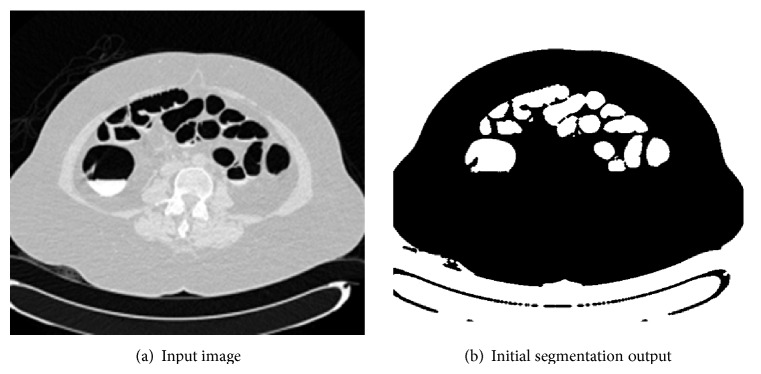
Extraction of air packets.

*Step 1: Extraction of Air Packets*. In this section, the input slice is considered, which comprises air packets. The air packets are removed using OTSU thresholding. Threshold selection of the input slices is based on OTSU thresholding which was introduced in the year 1979 [15]. OTSU method performs clustering by means of automatic thresholding and reduces the image from gray scale to binary form, through which the simple segmentation of image is processed. Initially the histograms of an image along with its probabilities are calculated for each and every unit in the slice. Thus this process separates the air and nonair regions. Through this a class variance is estimated using class probability and class mean of each unit in an image. An optimum threshold value will be selected based on the histogram in order to reduce the separability of two classes; hence the two foreground classes and the background classes are separated correctly so that intraclass variance is minimum. Thus the intraclass variance is the variation of pixel values within the same class, which will not vary to a large extent. The pixels corresponding to the air-filled colon are easily found as a result of this thresholding, often other air-filled anatomies like the lungs, small bowel, and stomachs, as well as the air outside the body, are included as in Figure 2 for a sample slice. Removal of these noncolon segments is described in the next section. The initial segmentation is denoted as IS. This initial step gives a rough approximation of the location of the colon. The other alternative techniques that can be used are *K*-means and Fuzzy *c* means clustering [16, 17].

The resultant image after extraction of air fluid packets approximately is shown in Figure 2. This process gives an approximate location of the colon.

*Step 2: Extraction of Lungs*. This module of operation deals with detecting and extracting boundaries from a binary transformed image. This module is used for tracing the outermost object (parent) and the holes which reside inside the outermost object (child). These modes are also capable of detecting parent and child objects in the binary image. This algorithm works based on Moore-Neighbor tracing algorithm modified by Jacob's stopping criteria [18, 19]. Thus the number of boundaries and the number of holes inside the boundaries are calculated. Thus, this step will generate the location of the boundaries [*B*] and number of continuous segments in each slice [*L*].

**Figure 3:**
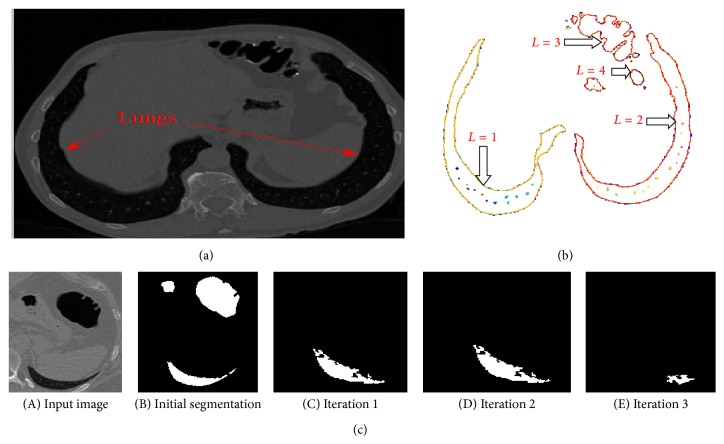
(a) Sample input slice. (b) Evaluation of continuous segments (*L*). (c) Results of extraction of lungs.

Step 2 is carried out to remove the lungs for the initial slices in which the coverage area of the lungs is more. The parameters considered for the removal of lungs are the specific area and the number of continuous regions in each slice (*L*). The various areas in the slices are analyzed by means of varying its threshold region. This process starts with the assignment of threshold value *L* = 1. For the initial slices, the segments containing the lung tissue will be more and the number of continuous regions will be minimum; hence “1” is chosen as the threshold value.  Figure 3, which has been considered for explanation, has value *L* = 4. There are 4 continuous or connected regions and the major portion of the slices occupies the lung tissue. Each connected component is uniquely labelled, which help in locating the organ of interest. Connected component labelling is used to detect connected regions in binary digital images. Connected component labelling works by scanning an image, pixel by pixel (from top to bottom and left to right) in order to identify the connected pixel region, that is, regions of adjacent pixels which share the same set of intensity values. Connected component analysis is performed using 8-neighbourhood connectivity. Thus the neighbouring pixels in all 8 eight directions are checked for connectivity.

After performing the labelling process, the different properties related to shape of the organs such as Area, Euler Number, Orientation, Bounding Box, Extent, Perimeter, Centroid, Extrema, and Convex hull are checked for each connectivity region. The parameter considered for the segmentation of abdominal organ is “area.” The area of all the pixels in an image is calculated by summing the areas of each pixel in the image. The specific area of the pixels occupied by the lungs will vary depending on the slice. The area coverage of the lung segment will be more for initial slices and will decrease for consecutive slices. Thus the maximum number of connected regions (*L*) that is to be considered for the segmentation of lungs was 18, because as the colon segments starts originating, the segments covered by the lung tissue will decrease; hence the number of continuous regions (*L*) will keep on increasing. For the slices which have the connected region *L* > 18, the above steps were not executed because the probability of occurrence of the lung segments will be minimum and more numbers of colon segments will start originating.

Thus, based on the above described process, lung regions are removed as shown in Figure 3(c) for various iterations when the area of lungs is small compared to the colon segments. For each iteration the connected segments corresponding to the lung are identified and finally it is removed as shown in the iteration 3 of Figure 3(c).

Based on the above feature, the resultant image *f*(*x*, *y*) will produce an output with a value of 1 where the organ is segmented and a value of zero elsewhere. Hence the lung region is extracted here from the resultant slice and compared with colon segments.

*Step 3: Morphological Processing*. The segmented slices of the above step will be followed by a morphological operation. This operation performs the morphological process by opening the single plane or gray scale image structurally. This process involves image erosion followed by dilation which leads to the smoothening inside the object. It opens the image object by means of reference structural elements specified by some required size and shape. There are two types of structuring elements involved which are flat and nonflat. In this work, the structuring elements used will be flat type such as shapes like disk with a radius of 2 units. This particular structuring element was used because the segment of the colon will approximate to the size of disk. Then, air-fluid boundaries are to be identified and extracted using efficient machine learning approaches to analyze and predict the exact form of tissue types air, liquid, soft tissue, muscle, and bone. Cascade feed forward back propagation neural network is proposed to extract the individual fluid packets which is discussed in next step.

**Figure 4:**
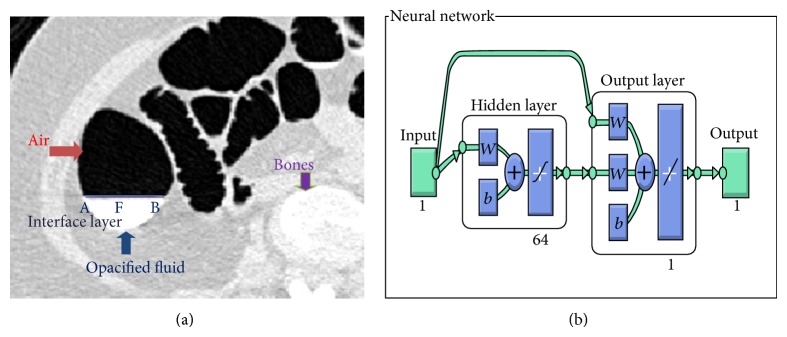
(a) Sample slice with partial volume effect. (b) Architecture of the training network.

*Step 4: Extraction of Individual Fluid Packets*. After external air and the preliminary process for the removal of lungs are completed, the identification and extraction of air-fluid boundaries are performed by using the concept of machine learning. In the CT images there are five tissue types air, liquid, soft tissue, muscle, and bone [20]. Figure 4(a) shows one transverse slice which is enhanced with opacified fluid.

The prior training of the partial volume effect region has been performed by using cascade feed forward back propagation neural network. Pattern association problem involves storing a set of patterns in such a way that when test data are presented, the pattern corresponding to the data is recalled and matched accordingly. The cascade feed forward neural network as shown in Figure 4(b) consists of input layer, hidden layer with 64 neuron, and 1 output layer. The input processed from the input layer is added with the weight; each subsequent layer consists of weights coming from the input and all previous layers. All layers have biases. Each layer's weights and biases are initialized. In experimentation, the numbers of neurons in hidden layer are increased from one neuron to 64 neurons. The procedure for the training of the neural network is performed by using the following procedure.

1. In this algorithm, a pattern is presented at the input layer. The neurons at this layer pass the pattern activations to the next layer neurons, which is actually the hidden layer. The outputs at the hidden layer neurons are generated using a threshold function along with the activations determined by the weights and the inputs. The threshold function is computed as 1/(1 + exp⁡(−*x*)) where *x* is the activation function value which is computed by multiplying the weight vector with the input pattern vector.
2. The hidden layer outputs become input to the output layer neurons, which are again processed using the same saturation function.
3. The final output of the network is eventually computed by the activations from the output layer.
4. The computed pattern and the input pattern are compared and in case of discrepancy, an error function for each component of the pattern is determined, and based on it the adjustments to weights of connections between the hidden layer and the output layer are computed. A similar computation, still based on the error in the output, is made for the connection weights between the input and hidden layers. The procedure is repeated until the error function reaches the range of the error tolerance factor set by the user.
5. The advantage of using this algorithm is that it is simple to use and well suited to provide a solution to all the complex patterns. Moreover, the implementation of this algorithm is faster and efficient depending upon the amount of input-output data available in the layers.

This theorem uses the knowledge of the prior events to predict the future events; hence this algorithm can be applied to our method and the network is trained a priori for the detection of partial volume effect and can be called in the future for the detection of partial volume effect in any dataset. Our results reveal that there is a tradeoff between number of neurons in hidden layer, learning time, and classification accuracy.

**Figure 5:**
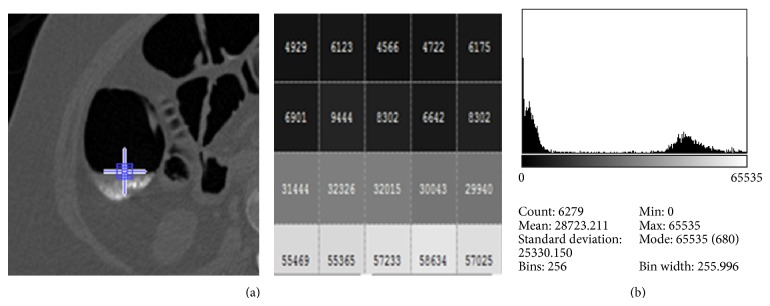
(a) The value of the pixels corresponding to the small blue square in the interface layer AFB. (b) Detailed histogram plot of the portion of the sample slice in (a).

The extraction of feature such as the intensity and the location was done by correlating the input slice with its corresponding ground. Based on the pixel values corresponding to the pattern of the partial value effect as shown in Figure 5(a), the assignment of the 16-bit pixel values for the different category of tissues was made as listed below.

 Assign
   A = 0 to 10000
     ∖∖ for intensity values from 0 to 1000 it is considered as region of colon filled with air;
   P/M = 30000 to 55000
     ∖∖ for intensity values from 1000 to 55000 it is considered as either muscles or a PVE;
   M = 10000 to 55000
     ∖∖ for intensity value from 10000 to 55000 it is considered as muscles;
   HI = 55000 to 63000
     ∖∖ for intensity values from 55000 to 65000 it is considered as either bones or liquid.

During training process, we took 7 different slices for analysis. The neural network was trained based on the following condition. Here true mentions the active region for extraction and false represents the region that need not be extracted.

The target will be true for the existence of Category A, Category A and P/M, and Category A, P/M and HI.

The target will be false for the existence of Category M, Category A and M, Category HI, and Category P.

1. The high intensity (HI) region which indicates both bone region and the liquid region lies in the intensity value from 55000 to 63000 so the only option to find the liquid region is that it lies near PVE and in the best case HI lies with both PVE and air region.
2. Are per our previous point discussion if HI or M or P intensities are found in a window alone then it is considered as bone and muscle and cannot be considered for colon or liquid or PVE.
3. If air region and PVE/muscle occur together without high intensity area then it is said to be inactive region.

**Figure 6:**
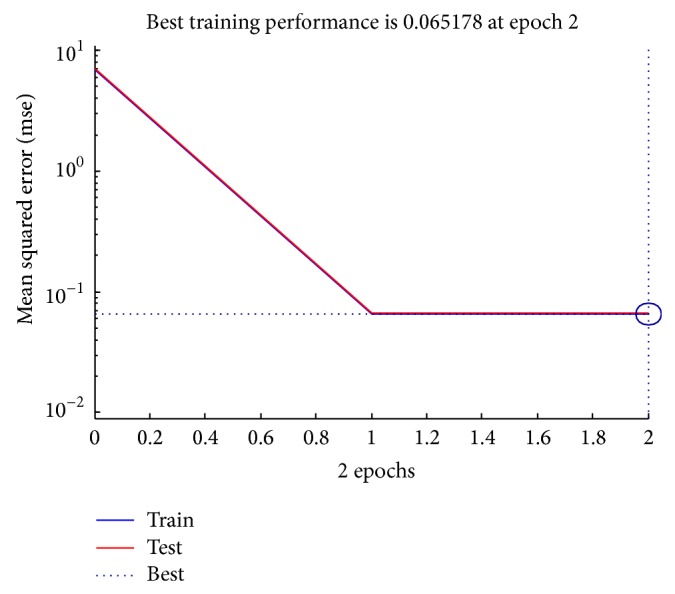
Performance plot.

The efficiency of the identification of the partial volume effect is evaluated with two parameters mean square error (MSE), the average squared error between the network outputs and the target outputs, and regression. The performance in the training window for a particular dataset is shown in Figure 6, a plot of the training errors and test errors. During training the weights and biases of the network are iteratively adjusted to minimize the network performance function. In this example, the result is reasonable because of the following considerations. The final mean-square error is small. The train and test set error have similar characteristics. No significant overfitting has occurred by iteration 2 (where the best validation performance occurs).

**Figure 7:**
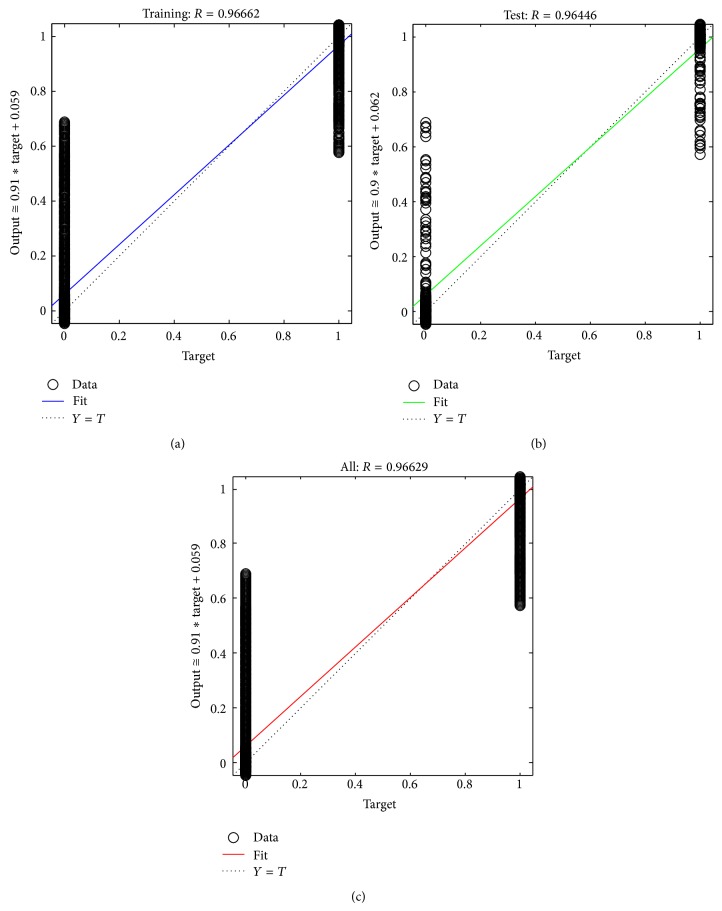
Regression analysis plot.

Regression analysis is a technique where the output will track the input and the value of 0.9 is a reasonable value as shown in Figure 7. Also the complete process is done with reduced time complexity, since a computer tomography image with hundreds of slices is used; the parameter mentioned in Figures 6 and 7 is much needed. Thus, the fluid packets are identified and extracted by the proposed neural network algorithm. Finally the colon is identified clearly and segmented by the following process.

*Colon Segmentation*

**Figure 8:**
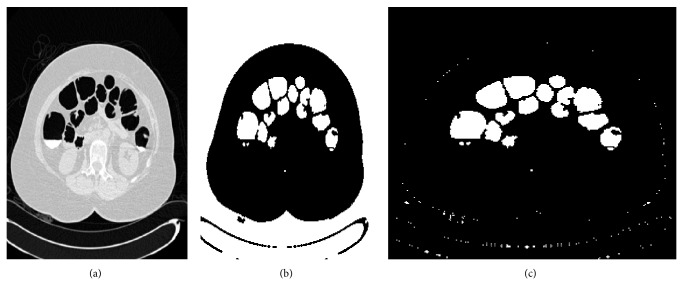
(a) Input: sample axial slice. (b) Output: *D*_0_. (c) OutF = And(*D*_0_, *D*_1_).

*Steps 5, 6, and 7: Performing AND Operation between D*_0_ *and D*_1_. For colon segmentation, the parallel entity to this process, the morphological open process (**I**_**mo**_), performed in Step 3 is divided into two branches, branch A and branch B. Branch A performs OTSU based binary transformation for the morphological open image **I**_**mo**_, which is denoted as *D*_1_. The output *D*_1_ for the sample input slice shown in Figure 8(a) is shown in Figure 8(b). At the same time branch B is processed in which the **I**_**mo**_ is let into already trained neural network object. The training for the network is provided by means of previously analyzed slice with partial volume effect mapped with its required ground truth formed by manual visual segmentation. The procedure for the training sequence is mentioned in the neural network training process. This output is denoted by *D*_0_. Now logical AND operation is performed for *D*_0_ and *D*_1_ known as OutF which is shown in Figure 8(c). This and operation is mainly performed to remove the background and segment the portions that are inside the body of the subject. Even though there are certain tiny holes, those holes could be removed by dividing the image into blocks.

**Figure 9:**
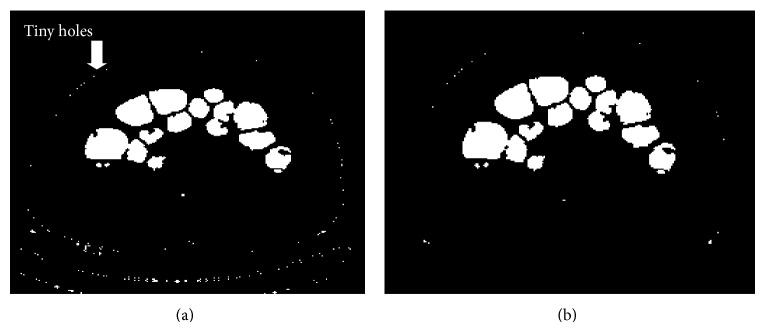
(a) OutF = And(*D*_0_, *D*_1_). (b) Removal of tiny holes.

*Step 8: Removal of Tiny Holes*. The segmented outputs produced by the previous step consist of tiny holes which are to be removed. The segmented image shown in Figure 8 which is of the resolution 512 × 512 is further divided into blocks where each block will be of the size 8 × 8; thus it will consist of 64 pixels. Thus a single slice is divided into 64 × 64 blocks and each block will consist of 64 pixels. The scanning of each block is done from left to right and from top to bottom. The area of the pixels is calculated for each block and compared with a threshold value of 10 to remove the small dots which prevail at the intersection of the foreground and the background. The steps that are followed for the removal of tiny holes are illustrated in Algorithm 1 and the corresponding output is shown in Figure 9(b). Thus 90% of the holes are removed.

Algorithm 1 results in removal of tiny holes in the slice. Then that slice is considered for AND operation.

*Step 9: Performing AND Operation*. The segmented output which is free of the tiny holes is undergone AND operation with the original segmented output (OI) that is performed in Step 2. The above Step 8 is performed only if the slices contain lungs. If the slices do not contain lungs the background is easily separated by setting all the pixels in the rows 1 till 40 and 400 till 512 to zero. Thus the unwanted colon parts can be removed. Let it be represented as OutF1. Finally, the bowels can be removed by performing slice difference removal method (SLDR).

**Figure 10:**
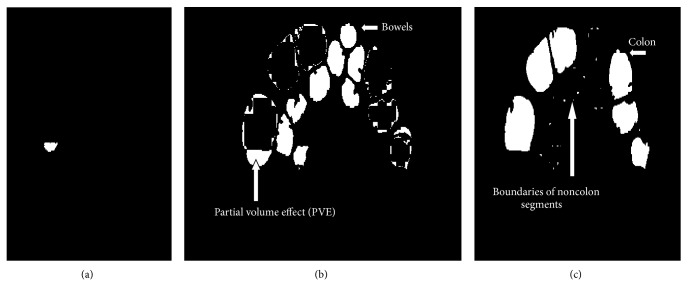
(a) Removal of PVE. (b) Removal of bowels. (c) Output of SLDR.

*Step 10: Removal of Bowels by Slice Difference Method*. In general, removal of bowels is a tedious process. SLDR is used in this work for removal of bowels without affecting the colon region. This method is evaluated by considering the segmented output of the previous slice and the current slice under consideration. The previous slice output is considered here as reference for the removal of bowels since the continuity exists from one slice to another slice. By undergoing an XOR operation between the previous segmented slice and the current segmented slice, the output will be zero in areas of colon as similar areas exist and high in areas of bowels as shown in Figure 10(b).

The operations are performed for each segment. Let the segments in a particular slice be represented as *X*_*i*_, *i* = 1,2,…, *n*. The algorithm is listed as shown in Algorithm 2.

The steps in Algorithm 2 will remove the bowels as shown in Figures 10(b) and 10(c), so this step will contain colon segments and some boundaries of noncolon segments.

This step is followed by the removal of the unwanted boundaries of noncolon segments as illustrated in Step 8; hence the images are further divided into blocks and scanned from left to right but this time the threshold is set to a value of 35. Thus the concatenation of the extracted PVE region shown in Figure 10(a) will be combined with the segmented colon segments to get the final output.

## 3. Results

**Figure 11:**
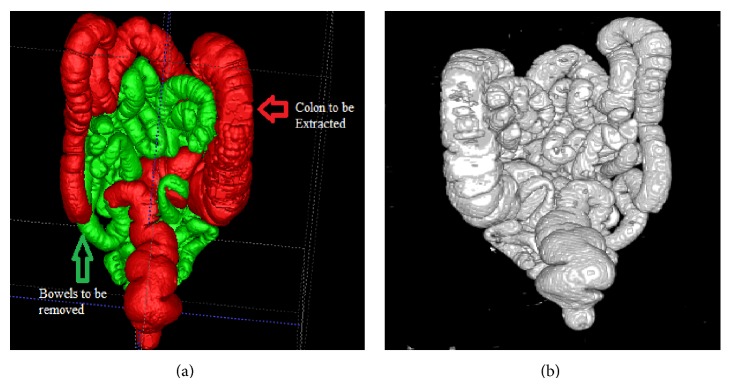
(a) Manual segmentation of dataset 1 which contains both bowels and colon. (b) Results of the automatic segmentation before the removal of bowels.

The experimental results are carried out to evaluate the performance of the proposed approach. The proposed segmentation method is used for segmentation of colon and this will serve as a basis for the identification of polyps which will lie on the walls of the colon for the diagnosis of colorectal cancer. For experiments, from the total of 20 datasets, 12 datasets were downloaded from TCIA cancer imaging archive, 6 in the supine position and 6 in the prone position. The remaining 8 datasets were real time datasets obtained from clarity imaging centre, Coimbatore. Thus the manual segmentation of dataset 1 segmented using ITK snap is shown in Figure 11(a).  Figure 11 contains colon, small intestine, mixed (of colon and small intestine through opened ileocecal valve which happens in low frequency), stomach or lung, and noise. Thus the colon portions that are to be segmented by the proposed method are highlighted in red colour and the bowels that are to be removed are highlighted in green colour. The results of the automatic segmentation generated by our proposed method before the removal of bowels are also shown in Figure 11(b). Thus the two figures show similar characteristics.

An example of colon segmentation for a few sample slices for two sample dataset is shown in Table 2. The sample dataset 1 consists of 629 slices. Tables 2(a), 2(b), and 2(c) show the final result of our proposed method for few sample slices, 137, 185, and 277 slices of the dataset 1. Thus the sample slices 186 and 277 shown in Tables 2(b) and 2(c) consist of colon, bowels, and fluid region; thus the removal of the bowel from the sample slice is highlighted by red colour in the category difference and the concatenation of the opacified fluid with the colon is shown in the output section.

**Figure 12:**
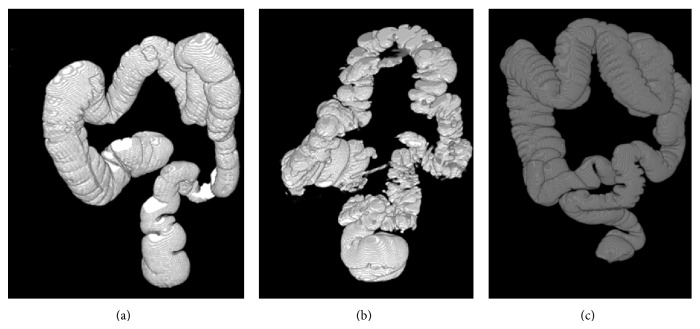
(a) 3D rendered output of dataset 1. (b) 3D rendered output of dataset 2. (c) 3D rendered output of dataset 3.

Then the output of sample axial slices 67 and 111 of dataset 2 (number of slices: 379) is shown in Tables 2(d) and 2(e). The 3D rendered outputs produced by the proposed segmentation method for sample three dataset are shown in Figure 12 when there is absence of polyps.

**Figure 13:**
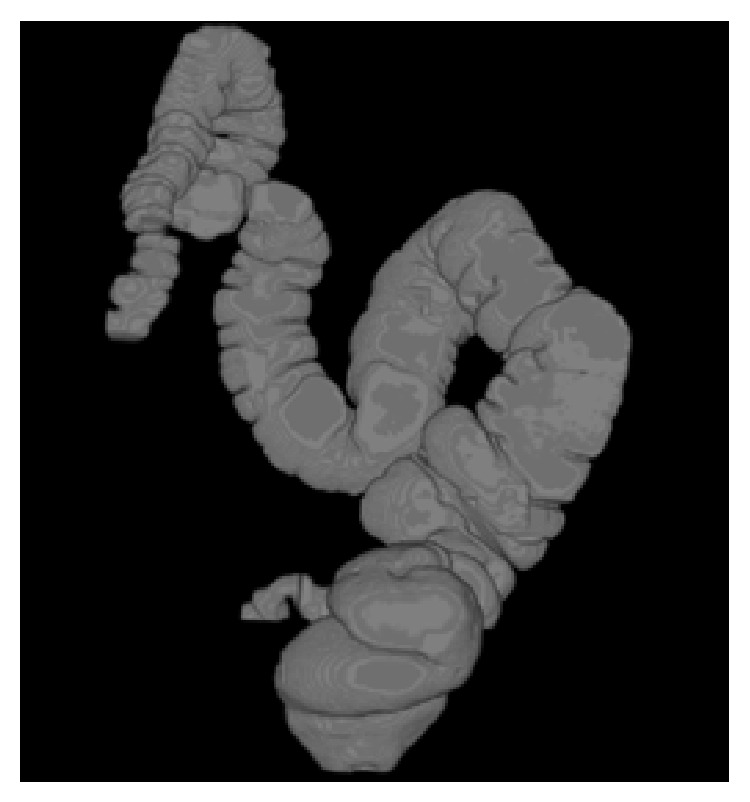
Segmentation of the colon when there is a polyp in the cecum.

If there is a polyp in the cecum then the segmentation of the colon will be incomplete as shown in Figure 13.

## 4. Discussion

The true positive fraction (TPF), or sensitivity, the specificity or false positive fractions (FPF), and accuracy which is the degree of closeness of measurements of a quantity to that quantity's actual-true value are also used for evaluation. For the evaluation of the above parameters, the number of colon segments which are classified correctly (true positives, TP) and misclassified as noncolon segments (false positives), the number of noncolon segments which are classified correctly as noncolon (true negatives, TN) and misclassified of colon segments (false negative, FN) should be calculated. In addition to the above parameters, the error and dice coefficient (DC) is also computed.  Table 3 depicts the average value of the TPF, FPF, DC, accuracy, and error. The equations that were used for the calculation of the parameters are as follows.

### 4.1. Sensitivity

The equation for the calculation of sensitivity is defined by

(1) $$\begin{array}{c} \text{Sensitivity}=\frac{\text{TP}}{\text{FN}+\text{TP}}. \end{array}$$

### 4.2. Specificity

The equation for the calculation of specificity is defined by

(2) $$\begin{array}{c} \text{Specificity}=\frac{\text{TN}}{\text{FP}+\text{TN}}. \end{array}$$

### 4.3. Dice Coefficient

To evaluate our method we compared its segmentation by our proposed method (B) with manual segmentations (gold standard) and computed the dice coefficient (DC) as a performance measure as specified in (3). If DC = 1 means perfect overlap between two segmentations [21, 22], then

(3) $$\begin{array}{c} \text{DC}=\frac{2\left|\text{A}\cap \text{B}\right|}{\left|\text{A}\right|+\left|\text{B}\right|}. \end{array}$$

### 4.4. Accuracy

The equation for the calculation of accuracy is defined by

(4) $$\begin{array}{c} \text{Accuracy}=\frac{\text{TP}+\text{TN}}{\text{FP}+\text{FN}+\text{TP}+\text{TN}}. \end{array}$$

The results of our proposed method were reported in Table 3 and in Table 4 results were compared with those of two other segmentations technique proposed for the segmentation of colon: adaptive level sets and graph cuts. In all the cases the results of the algorithms are compared with those obtained by the manual identification of the colon segments by radiologist.

Table 3 depicts the average value of the TPF, FPF, DC, accuracy, and error. From the table, it can be said that the proposed method performs better on several datasets in terms of sensitivity, specificity, accuracy, and so forth.

The algorithm correctly identified the large colon segments. All the false positive fractions were due to the small bowel which lies very close to the colon and is indicated in green color in Figure 11 and Table 2(b) in the category difference. The size, position, and the occurrence of bowels will decide the overall performance of the proposed method. The lungs segments which should be removed perfectly by the lung removal stage also decide the parameter values since the large segments are easily removed and care has been taken to remove even the small lung segments and still a fraction of the small lung segments result in the lowering the parameters value.

The accuracy of the graph cut approach is of a lower grade compared to that of the other two techniques, even though it has the benefit of finding global solution. This is unlike the level sets approach which gets straightforwardly trapped in local minima, requiring manual seeds to be placed inside every colon segment for the proficient segmentation process. Although level sets provide high segmentation accuracy which is close to the proposed method, we highlight that manual seeding is time consuming and error-prone. Thus our proposed method does not stuck anywhere and even very small colon segments which have been missed by the radiologist are correctly identified by our method.

The results of the proposed method are reported in Table 3; results were compared with those of two other segmentation techniques like adaptive level sets and graph cuts. In all the cases the results of the algorithm are compared with those obtained by the manual identification of the colon segments by radiologist. The number of slices per dataset varies from 350 to 650 depending on the height of the patient.

Table 4 shows the comparison results of existing and proposed method. The proposed segmentation method achieves 98% of accuracy in colon segmentation, whereas the Graph Cuts approach is 90.8% and Level Sets is 97.6%. This is due to efficient proposed approaches and their step by step process. Similarly, the sensitivity value of the existing Graph Cuts and Level Sets algorithm is of 94.1% and 96.02%, whereas the proposed segmentation approach acquires 96.75%. Specificity (sometimes called the true negative rate) measures the proportion of negatives which are correctly identified, in which the proposed approach specificity value is 97%, whereas the Graph Cuts approach is 94.3% and Level Sets is 97%. Hence the proposed approach performs better when compared to other existing approaches.

## 5. Conclusion

Accurate and automatic colon segmentation is crucial for a virtual colonoscopy system. The proposed approach was validated on the data downloaded from TCIA cancer imaging archive and on real data set. TCIA cancer imaging archive has a wide variety of collections of CT colonography, in which the 243 cases are free of polyps. The dataset that has been used here is among the 243 cases. The proposed method will segment different portions of colon for each slice using the steps which has been illustrated earlier and the segmented portions were compared with the ground truth images and the different parameters were calculated to validate the proposed method. Thus the proposed automatic segmentation method outperformed the other two methods as the importance is given to the extraction of opacified fluid and the removal of even small bowels that may lower the accuracy value. We provided quantitative results based on 20 datasets having challenges. Hence the proposed work obtains accurate results for a fully automated system, compared with other known segmentation algorithms.

## Figures and Tables

**Algorithm 1:**
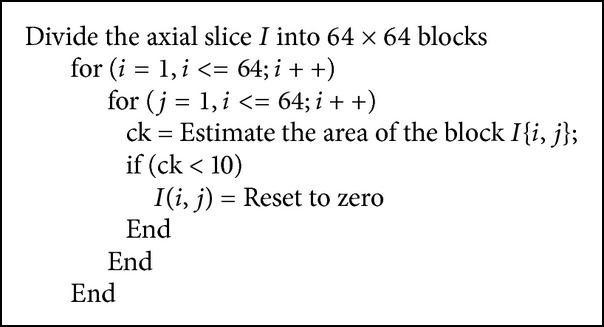
Steps implemented for the removal of tiny holes.

**Algorithm 2:**
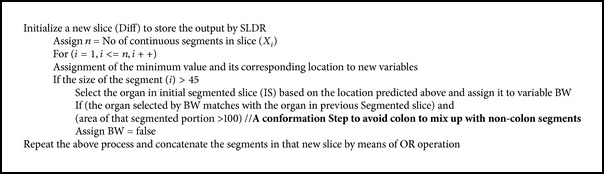
Steps implemented for the removal of bowels.

**Table 1:** Comparative study of the method proposed with its limitation.

Author	Method	Limitation
Bert et al. (2009) [5]	Automatic segmentation of colon using 3D seeded region growing algorithm	It is obvious that the most serious problem of region growing is the power and time consuming

Losnegård et al. (2010) [7]	Semiautomatic segmentation	Disadvantage is that it consumes more time and lesser accuracy

Lu and Zhao (2011) [8]	Noncolonic attachment classification algorithm and a heuristic connection algorithm	This method could achieve 92.86% coverage of human-generated colons, which is of 13.68% higher than the conventional method

Chowdhury and Whelan (2011) [9]	Automatic colon segmentation from CT data using colon geometrical features	This approach performs better and provides efficient results in colon segmentation

Kilic et al. (2009) [12]	Automatic three-dimensional computer-aided detection system	Average coverage is about 87.5% of the entire colon

Taimouri et al. (2011) [10]	Constrained least-squares filtering (CLSF)	Applicable only for specific cases, not converged early

**Table 2:** Output of sample axial slices of dataset.

Index	Input image	*D*_0_	Ground truth	Difference	Output
Sample slice number					
	Output of sample axial slices of dataset 1 (number of slices: 629)				
(a) 137	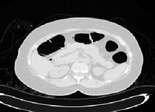	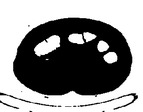	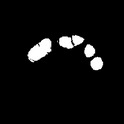	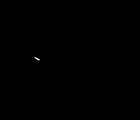	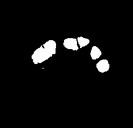
(b) 185	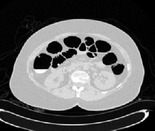	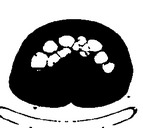	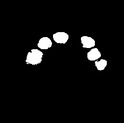	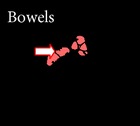	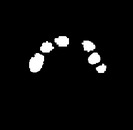
(c) 277	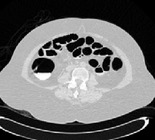	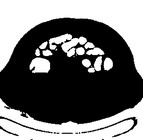	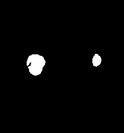	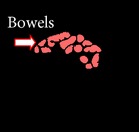	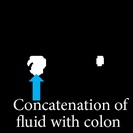

	Output of sample axial slices of dataset 2 (number of slices: 379)				
(d) 67	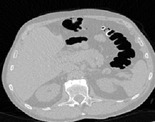	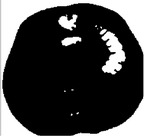	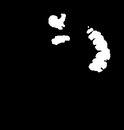	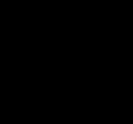	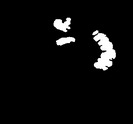
(e) 111	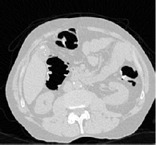	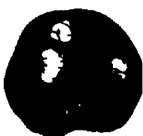	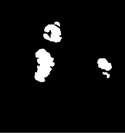	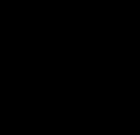	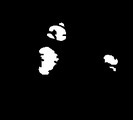

**Table 3:** List of parameters evaluated by the proposed method.

Number of patient	Specificity (%)	Sensitivity (%)	Dice coefficient (measured over value 1)	Accuracy (%)	Error (%)
12 datasets from cancer imaging archive					
10 well distended	98	99	0.97	98.1	2
2 collapsed	95	95	0.91	98	10

8 real time datasets					
5 well distended	98	98	0.96	98.2	2
3 collapsed	96	95	0.91	98	5
Average	**97**	**96.75**	**0.94**	**98**	**—**

**Table 4:** Tabulation of comparison of the parameters.

Parameters	Graph cuts [23]	Level sets [24]	Proposed method
Accuracy	90.8%	97.6%	98%
Sensitivity	94.1%	96.02%	96.75%
Specificity	94.3%	96.8%	97%
